# Evaluation of Arterial Stiffness in Depression Patients

**DOI:** 10.31083/AP46112

**Published:** 2025-08-28

**Authors:** Burcu Sırlıer Emir, Sevler Yıldız, Aslı Kazgan Kılıçaslan, Gülhan Kılıçarslan, Osman Kurt, Sevda Korkmaz, Murad Atmaca

**Affiliations:** ^1^Department of Psychiatry, Elazığ Fethi Sekin City Hospital, 23100 Elazığ, Turkey; ^2^Department of Psychiatry, University of Binali Yıldırım, 24002 Erzincan, Turkey; ^3^Department of Psychiatry, University of Bozok, 66200 Yozgat, Turkey; ^4^Department of Public Health, Adıyaman Provincial Health Directorate, 02400 Adıyaman, Turkey; ^5^Department of Psychiatry, University of Fırat, 23119 Elazığ, Turkey

Dear Sir,

We read with interest Saleh’s letter [1] commenting on our article published in Alpha Psychiatry [2]. We would like to make some clarifications regarding these reviews.

As outlined in the Mannheim Intima-Media Thickness Consensus, Intima-Media Thickness (IMT) should preferably be measured on the far wall of the common carotid artery (CCA), at least 5 mm below its end [3].

This avoids inter-individual variability triggered by physiologic remodeling and has less gain dependence. Values taken at the near wall are partly dependent on gain settings and are less reliable. If taken from the near wall, its value should be recorded separately from the IMT from the far wall. We took multiple measurements at least 5 mm before the bifurcation of the carotid artery (we took 1 cm before), from the far wall where the double line (intimal and medial line) was clearly visible and used the mean value in the calculations. We acknowledge that the omission of this detail in the methodology section is a shortcoming. Our inability to perform measurements with electrocardiogram (ECG) synchronization is due to the lack of equipment in our hospital. Diastolic measurements could have been performed with M mode, but the image is too small and the resolution is too low when both modes are working at the same time. Measurements can be made from the carotid bifurcation or internal carotid artery (ICA) bulb, taking into account the large interindividual variability caused by IMT, remodeling and anatomical variations. Since the ICA is tortuous in many cases, we preferred to measure before the bifurcation for standardization.

The reason why we did not measure from the near wall is that this section could not be clarified because of the calibration problems of our devices. It should be noted that, although this was not clearly reported in our report, both carotid artery and femoral artery measurements were made before the bifurcation. Measurements were made from the right and left sides, but we used unilateral measurements because there were patient discrepancies and there were articles in the literature with only right-sided measurements. There were articles in the literature with only right-sided measurements [4, 5].

## Availability of Data and Materials

The data that support the findings of this study are available on request from the corresponding author.
